# Substantial impact of FDG PET imaging on the therapy decision in patients with early-stage Hodgkin's lymphoma

**DOI:** 10.1038/sj.bjc.6601561

**Published:** 2004-02-03

**Authors:** R Naumann, B Beuthien-Baumann, A Reiß, J Schulze, A Hänel, J Bredow, G Kühnel, J Kropp, M Hänel, M Laniado, J Kotzerke, G Ehninger

**Affiliations:** 1 Department of Medicine I, University Hospital Carl Gustav Carus at the Dresden University of Technology, Fetscherstr. 74, 01307 Dresden, Germany; 2 Department of Nuclear Medicine, PET Center Rossendorf, University Hospital Carl Gustav Carus at the Dresden University of Technology, Fetscherstr. 74, 01307 Dresden, Germany; 3 Department of Diagnostic Radiology, University Hospital Carl Gustav Carus at the Dresden University of Technology, Fetscherstr. 74, 01307 Dresden, Germany; 4 Department of Haematology/Oncology, Clinic of Internal Medicine III, Chemnitz Medical Center, Buergerstr. 2, 09113 Chemnitz, Germany

**Keywords:** Hodgkin's lymphoma, ^18^F-fluorodeoxyglucose (FDG), positron emission tomography (PET), staging, lymphoma

## Abstract

This prospective study assessed the impact of ^18^F-fluorodeoxyglucose (FDG) positron emission tomography (PET) on the staging and possible consequential changes of treatment regimen in patients with Hodgkin's lymphoma (HL). A total of 88 consecutive patients with histologically verified Hodgkin's lymphoma underwent a PET scan in addition to conventional staging procedures. Treatment was based on the conventional staging only, and the results of the FDG-PET did not affect the treatment strategy. The evaluation focused on the suggested change in clinical stage according to the Ann Arbor classification and on the suggested change in treatment strategy rather than on a lesion-by-lesion analysis. Using all the methods performed as the standard of reference, ^18^F-FDG-PET staging was concordant with conventional staging in 70 out of 88 patients (80%). ^18^F-fluorodeoxyglucose positron emission tomography suggested a change to a different clinical stage in 18 patients (20%). Management would have been changed in 16 patients (18%): intensification of treatment in nine patients (10%) and minimisation of treatment in seven patients (8%). In the 44 patients with early disease (stage IA–IIB), treatment would have been intensified in nine out of 44 patients (20%). ^18^F-fluorodeoxyglucose positron emission tomography is a relevant noninvasive method that supplements conventional staging procedures and should therefore be used routinely to stage Hodgkin's lymphoma, particularly in patients with an early stage.

Today, Hodgkin's lymphoma (HL) is one of the malignant diseases with the highest rate of cure in adults based on improved chemotherapy and radiotherapy. The most important objective of staging is the exact detection of all nodal and extranodal lymphoma manifestations present to be able to administer the optimal therapy according to the stage and to the risk situation. The criteria for detecting involvement are mainly based on the assessment of the size of the lymph nodes, mainly revealed with computed tomography (CT), regarding lymph nodes with a diameter of more than 1 cm as pathologic. Although these criteria are generally accepted, it is known that on the one hand lymph nodes that are smaller than 1 cm may already have undergone malignant transformation and on the other hand, benign lymphadenopathy may also lead to enlarged lymph nodes (Castellino *et al*, 1984). Focal bone marrow involvement is typical in HL. It may be missed even if a bilateral bone marrow biopsy is taken in the dorsal iliac crest (sampling error) (Brunning *et al*, 1975).

As a noninvasive functional method, ^18^F-fluorodeoxyglucose (^18^F-FDG-PET) offers tomographic imaging and quantification of the metabolic activity of tumour tissue with a high spatial resolution. Supplementing the standard investigations, ^18^F-FDG-PET may provide further diagnostic information for staging. Today, the high sensitivity and specificity of PET for staging is no longer disputed (Kostakoglu and Goldsmith, 2000; O'Doherty *et al*, 2002; Schiepers *et al*, 2003; Staak *et al*, 2003). ^18^F-fluorodeoxy-glucose is the most frequently used PET tracer in the investigation of malignant lymphoma (Hustinx *et al*, 2003). The majority of published studies addressed the staging of lymphoma; only limited data on the impact of FDG-PET on the treatment planning are available.

The aim of this prospective study was to assess the clinical impact of ^18^F-FDG-PET on the therapeutic management in a larger patient group than has been published up to now.

## MATERIALS AND METHODS

### Patients

Between April 1997 and April 2002, 88 consecutive patients with primary (*n*=77) or recurrent (*n*=11) HL were enrolled in our prospective study at the Department of Medicine I, University of Dresden, and the Department of Haematology/Oncology, Chemnitz Medical Center, Germany. The disease was verified histologically in all patients using the World Health Organization (WHO) classification system (Harris *et al*, 1999). Patients were examined with both conventional staging procedures and PET imaging within 4 weeks before the initiation of therapy. The local ethics committee approved the study. All patients gave their written informed consent for the performance of PET, which was regarded as a routine procedure in staging. The patients characteristics are presented in Table 1

**Table 1:** Patient characteristics (*n*=88)

		**Patients**	
**Characteristic**		**No.**	**%**
*Sex*			
Male		57	65
Female		31	35

*Age (years)*			
Median		34	years
Range		17 to 83	years

*Disease status*			
Initial diagnosis		77	87
Recurrent disease		11	13

*Histology (WHO classification)*			
Classical HL			
Nodular sclerosis HL		52	59
Lymphocyte-rich classical HL		1	1
Mixed cellularity HL		29	33
Lymphocyte depletion HL		1	1
Hodgkin's but unclassified		3	3
Nodular lymphocyte-predominant HL		2	2

*Clinical stage* (conventional staging procedures)			
I/IE	7/1	8	9
II/IIE	33/3	36	41
III/IIIE	22/6	28	32
IV	16	16	18

HL=Hodgikn's lymphoma.

.

### Conventional staging procedures

The routine staging comprised physical examination, chest X-ray, ultrasound, contrast-enhanced CT of the neck, chest, abdomen and pelvis. Full laboratory work-up included erythrocyte sedimentation rate (ESR) as well as posterior iliac crest biopsy for bone marrow evaluation. Magnetic resonance tomography (MRI) or bone scan was performed additionally in individual patients or when there was suspicion of osseous involvement. Nodal involvement was defined as lymph node enlargement of 1 cm or more on the basis of clinical or imaging findings. The clinical stage of the patients was assessed according to the Ann Arbor classification (Carbone *et al*, 1971).

### ^18^F-FDG-PET imaging

^18^F-fluorodeoxyglucose positron emission tomography was performed from the proximal femur to the base of the skull and, depending on the involved locations, also on the head and lower extremities. The patients fasted for at least 6 h before the PET scan. At 45–60 min after injection of 300–370 MBq ^18^FDG, the investigation was carried out with an ECAT EXACT-HR+ scanner (Siemens/CTI, TN, USA) with an axial field of view of 15.5 cm. The spatial resolution was 4.0 mm (axial) and 4.2 mm (transaxial). Six bed positions were taken for 8 min each. A transmission scan for attenuation correction was obtained for each patient using rotating ^68^Ga/^68^Ge rod sources. Coronal, sagittal and transverse data sets were reconstructed. The findings were interpreted visually after examining the section in the black and white mode on a high resolution display by at least two experienced investigators in consensus. Pathologically raised FDG uptake was analysed quantitatively using regions of interest (ROIs) and by determination of the standardised uptake values (SUVs) (Strauss and Conti, 1991). Regions of interest were marked with isocontours around a suspicious lesion, setting the lower threshold at the level of SUV 2.0.

### Treatment protocols

Patients with primary diagnosis were assigned to an early, intermediate and advanced stage according to the current risk factors of the German Hodgkin's Lymphoma Study Group, GHSG (Sieber *et al*, 2000). This risk stratification may entail a change in therapy despite identical stage due to the presence of an additional risk factor (Table 2

**Table 2:** Treatment protocols for patients with primary diagnosis (*n*=77)

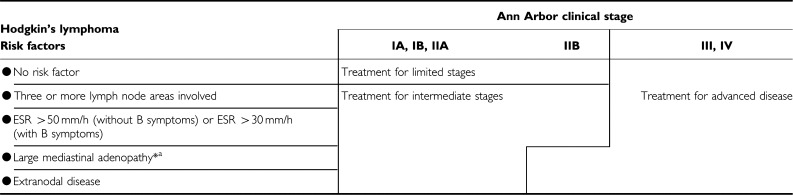

). Between April 1997 and April 2000, the patients were enrolled in the protocols of the GHSG. In the limited stage, the patients received either extended field (EF) irradiation alone or ABVD plus EF irradiation (trial HD 7 of the GHSG). In the intermediate stage, the patients were treated with two cycles of COPP-ABVD with EF or involved field (IF) irradiation (trial HD 8). In the advanced stage, the patients obtained COPP--ABVD or the BEACOPP regime (trial HD 9). Between May 2000 and April 2002, patients were treated in pilot studies using identical inclusion criteria. In the limited or intermediate stage, the patients received four or six cycles of ABVD, respectively, plus IF irradiation in case of residual mass (more than 1.5 cm in CT). In the advanced stage, the patients were treated with the etoposide-free BACOPP-D regimen, which included cyclophosphamide, adriamycin, dacarbazine, procarbazine, prednisolone, bleomycin and vincristine (Naumann *et al*, 2002b). In cases of recurrence in patients with stage I or II disease, the question of the feasibility of curative irradiation was considered. In patients in whom it was not feasible, as well as in the stages III and IV, salvage chemotherapy and if necessary high-dose chemotherapy with stem cell support was administered.

### Standard of reference

As pathologic confirmation of all lesions (gold standard) was impossible, the results of all methods performed were subsumed in a standard of reference. Positive findings at clinical examination, CT or another conventional technique and appropriate ^18^F-FDG-PET findings were interpreted as a manifestation of HL (true-positive). Negative findings with both standard methods and ^18^F-FDG-PET were regarded as true-negative. The result of the comparison between conventional diagnostics and ^18^F-FDG-PET was rated as concordant if both procedures led to an identical clinical stage. In cases of discrepancy, imaging studies were reviewed by an interdisciplinary panel comprising of colleagues from the departments of nuclear medicine, diagnostic radiology and haematology. In these patients, PET imaging findings within final staging as well as response to therapy and follow-up information were used to evaluate the precision of the initial PET scans.

## RESULTS

All patients could be assessed for evaluation.

### Concordant results

Using all the methods performed as the standard of reference, staging by ^18^F-FDG-PET was concordant with conventional staging procedures in 70 out of 88 patients (80%). Of these 70 patients, six had stage I, 29 had stage II, 20 had stage III and 15 had stage IV according to the Ann Arbor classification.

### Discordant results

In 18 patients (20%), discordant results between PET and conventional staging procedures were found. *True-positive PET findings:* In 11 patients (13%), additional lymphoma sites were found. *True-negative PET findings:* In one patient, a false-positive cervical ultrasound finding was correctly identified by PET. *False-negative PET findings:* In three patients, ^18^F-FDG-PET could not identify retroperitoneal nodal involvement. In one patient, ^18^F-FDG-PET did not detect biopsy-proven liver involvement. ^18^F-FDG-PET failed to detect inguinal and cervical nodal HL in two more cases. Response to therapy and final CT scans were used to verify the described false-negative nodal PET findings in retrospect. *False-positive PET findings:* No false-positive PET findings occurred.

### Change in clinical stage

**Figure 1:**
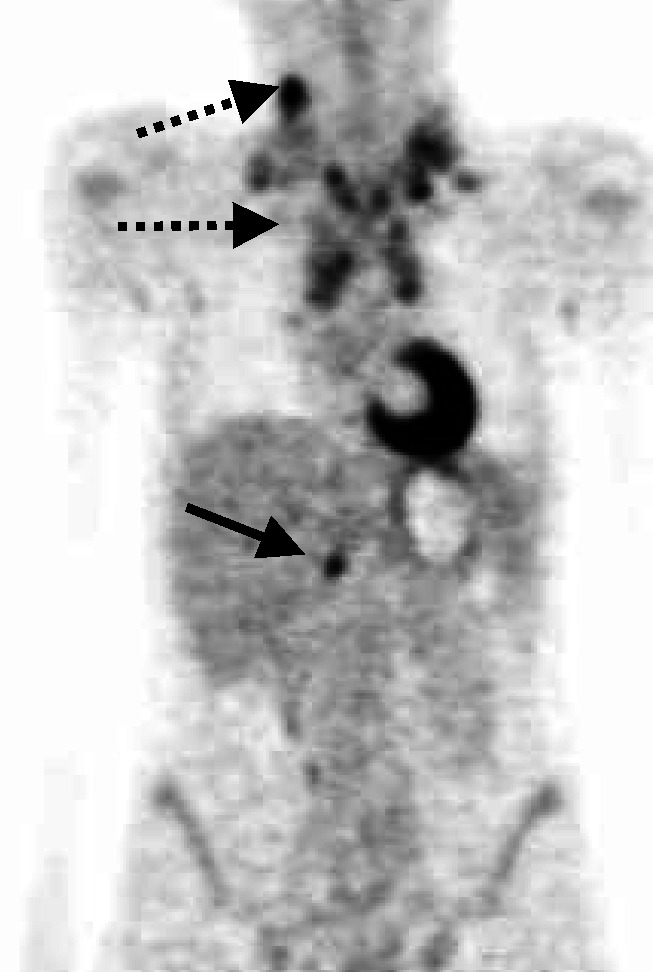
Additional focal liver uptake (solid arrow) would have changed former stage II (cervical and mediastinal involvement, dashed arrows) to stage IIIE (patient no. 13, Table 3).

Of the 88 patients examined, the staging would have changed in 18 patients (20%) as a result of the ^18^F-FDG-PET scan. In all, 11 patients (13%) would have been upstaged and seven patients (8%) would have been downstaged. There would have been one upstaging from clinical stage I to II, one from I to IV, four from II to III, one from II to IV and four from III to IV. Figure 1

shows the PET image of one patient, where the additional focal liver uptake would have changed the stage from II to IIIE according to the modified Ann Arbor classification (patient no. 13). Downstaging was suggested for two patients from II to I, one patient from III to I, three patients from III to II and one patient from IV to II.

### Change in treatment strategy

The treatment strategy would have been identical in 72 of 88 patients (82%). As a result of change of stage and risk factors (Table 2), the treatment strategy would have been changed in 16 of 88 patients (18%). In a total of nine of the 88 patients (10%), treatment would have been intensified: as a result of upstaging in six patients and due to an additional risk factor (three or more lymph node locations involved) in the other three patients. Three of the 11 patients with recurrent disease would have been upstaged with consecutive high-dose chemotherapy with autologous stem cell transplantation. The evaluation of the 44 patients in stage I and II after conventional staging would have resulted in intensification of therapy in nine patients (nine out of 44, 20%). Focusing on the 36 patients with stage II disease, ^18^F-FDG-PET would have led to intensification of treatment in more than one-fifth of the patients (eight out of 36, 22%). One patient (1%), who would have been downstaged, would have received reduced therapy within the scope of the treatment group comprising patients with limited stages. The further six patients with false-negative results would have been downstaged with the consequence of treatment minimization.

**Figure 2:**
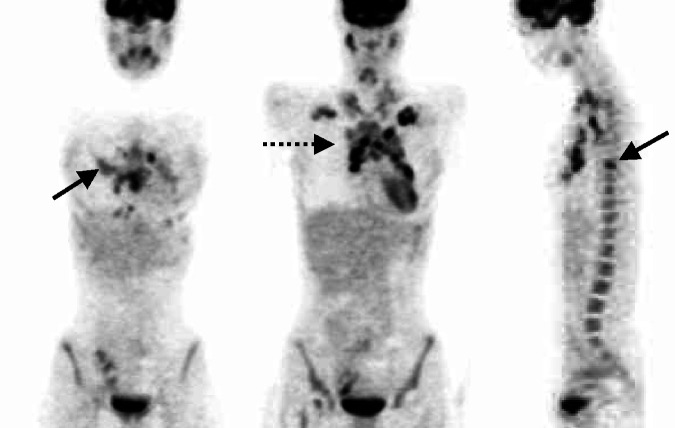
Besides the known infraclavicular and mediastinal uptake (dashed arrow), the multifocal bone marrow uptake (solid arrows) was only detected by FDG-PET (patient no. 14, Table 3).

In six patients, ^18^F-FDG-PET findings would have resulted in upstaging to a stage IV. In two patients, disseminated osseous involvement (patient no. 2) or bone marrow involvement (patient no. 14) with consequences for therapy were diagnosed using supplementary ^18^F-FDG-PET. The upstaging from stage III to stage IV would not have translated into changes in treatment strategy in four patients, including one patient with liver involvement (patient no. 4), one patient with osseous involvement (patient no. 21) and two patients with liver and osseous involvement (patient no. 16, 20). Infiltration of the bone marrow was diagnosed in three patients. In two patients, concordant results were obtained from bone marrow biopsy and ^18^F-FDG-PET. As mentioned above, in one woman (patient no. 14) bone marrow involvement that would have affected the treatment strategy was detected only by PET. A focally raised FDG uptake could be detected at multiple locations in this patient (Figure 2

) that could no longer be demonstrated at post-treatment follow-up. Table 3

**Table 3:** Patients with suggested changes to stage and/or treatment strategy due to ^18^F-FDG-PET

		**Stage**			
**Patient no.**	**Disease status (P, R)**	**Standard**	**PET**	**Change in stage**	**Change in treatment**
1	P	IIIA	IIA	Downstaging	Minimisation
2	R	IAE	IVA	Upstaging	Intensification
3	P	IIIB	IIB	Downstaging	No change
4	P	IIIA	IVA	Upstaging	No change
5	R	IIAE	IIIAE	Upstaging	Intensification
6	R	IIB	IIIB	Upstaging	Intensification
7	P	IVA	IIA	Downstaging	Minimisation
8	P	IIIA	IA	Downstaging	Minimisation
9	P	IIA	IIIA	Upstaging	Intensification
10	P	IIIAE	IIAE	Downstaging	Minimisation
11	P	IIA	IIAE	No change	Intensification
12	P	IIB	IB	Downstaging	Minimisation
13	P	IIA	IIIAE	Upstaging	Intensification
14	P	IIA	IVA	Upstaging	Intensification
15	P	IA	IIA	Upstaging	No change
16	P	IIIB	IVB	Upstaging	No change
17	P	IIA	IIA	No change	Intensification
18	P	IIA	IIA	No change	Minimisation
19	P	IIA	IA	Downstaging	Minimisation
20	P	IIIB	IVB	Upstaging	No change
21	P	IIIB	IVB	Upstaging	No change
22	P	IIA	IIAE	No change	Intensification

P=primary diagnosis, R=recurrent disease.

shows the 22 patients with discrepant findings after conventional (standard) staging procedures and ^18^F-FDG-PET.

## DISCUSSION

Precise staging is prerequisite to establish the optimum treatment strategy in patients with HL (Lister *et al*, 1989). Owing to the increasing use of combination chemotherapy at early stages, the result of the H6-F study of the EORTC, use of prognostic factors and improved CT and MRI techniques, staging laparotomy has disappeared as a routine staging procedure (Glatstein *et al*, 1969; Carde *et al*, 1993).

In the last few years, the value of FDG-PET for the staging of malignant diseases has been recognised. The use of elevated ^18^F-FDG uptake as an indicator for malignant disease renders ^18^F-FDG-PET independent of morphological conditions. The published sensitivity and specifity emphasises the value of ^18^F-FDG-PET in the staging of lymphoma and explains the increasing acceptance of this method (Jerusalem *et al*, 2001; Naumann *et al*, 2001; Spaepen *et al*, 2001; Kostakoglu *et al*, 2002; Guay *et al*, 2003). Elstrom *et al* were able to confirm the high sensitivity of ^18^F-FDG-PET for HL, diffuse large-cell NHL, and for the follicular subtype of indolent NHL (Elstrom *et al*, 2003).

A review of the literature only revealed a small number of studies concentrating solely on primary and recurrence staging in HL. Numerous studies were retrospective, being based on different lymphoma classifications and investigating ‘malignant lymphomas’ without specific analyses of HL and the heterogeneous group of NHL. Earlier reports referred above all to the excellent precision of ^18^F-FDG-PET. It led to upstaging or downstaging with a high sensitivity and specificity in 10–20% of patients. However, only a slight effect on the choice of treatment was stated. Our own data obtained in quite a large number of patients unequivocally documented the therapeutic relevance of ^18^F-FDG-PET for patients with HL.

The prospective study of Bangerter *et al* (1998) entailed a change in treatment in six of 44 patients (14%). Jerusalem *et al* (2001) investigated 33 patients with HL. Upstaging was indicated in three patients (9%) and downstaging in four patients (12%). However, this did not translate into changes in treatment strategy (Jerusalem *et al*, 2001). In the study of Hueltenschmidt *et al* (2001) there was a change in treatment in two cases (8%), one of which entailed intensification and one a minimisation of treatment. In the prospective study of Menzel and colleagues, a correct change in stages resulted in six out of 28 (21%) patients (Menzel *et al*, 2002). Weihrauch *et al* (2002) reported on upstaging in four patients out of a total of 22 patients investigated (18%). Of these, one patient underwent consequent intensification of treatment in accordance with the therapy strategies of the GHSG (Weihrauch *et al*, 2002). The rate of upstaged patients in our study (13%) was quite similar.

To our knowledge, our study comprising 88 prospectively investigated HL patients is the largest-scale PET study on this topic that has been published up to now. The main result of our study was the high rate of treatment intensification in patients with limited stages I and II (20%). The selective consideration of patients with stage II increased the rate to 22%.

False-negative PET findings in the detection of CT-positive and PET-negative areas underscore our own observation and our recommendation to use ^18^F-FDG-PET not instead, but in combination with conventional diagnostics. In six patients (7%) of our study, the evaluation using the standard of reference prevented a downstaging owing to false-negative PET findings. In a further female patient, the ^18^F-FDG-PET did not detect a third and thus therapy-relevant lymph node location. Theoretically, in a total of six patients (7%) this would have erroneously led to a minimisation of therapy. Weihrauch *et al* (2002) reported a similar experience in their prospective study mentioned above, in which ^18^F-FDG-PET missed Hodgkin's foci in six patients (27%). Although inflammatory lesions, in particular, can cause a pitfall in PET imaging, we did not observe any false-positive PET findings that suggested erroneous upstaging in our study (Moog *et al*, 1998a; Hueltenschmidt *et al*, 2001; Naumann *et al*, 2002a).

In our study, ^18^F-FDG-PET suggested upstaging into a clinical stage IV in six cases, in two patients with consequences for treatment owing to the detection of disseminated bone involvement and bone marrow infiltration (BMI). In fact, ^18^F-FDG-PET is more sensitive and specific than bone scintigraphy in identifying cortical bone involvement in malignant lymphoma and is consequently likely to supersede this method (Moog *et al*, 1999). ^18^F-fluorodeoxyglucose positron emission tomography as a sensitive method for the detection of bone marrow infiltration can provide additional information to that of bone marrow biopsy in the staging of malignant lymphomas (Bangerter *et al*, 1998; Carr *et al*, 1998; Moog *et al*, 1998b; Naumann and Beuthien-Baumann, in press).

On the basis of our results, we consider ^18^F-FDG-PET to be of value especially in stages I and II, since in these stage groups the input from the PET result on the treatment was substantial. In stages III and IV, ^18^F-FDG-PET may not be cost effective. As in most other publications, our investigation was a monocentric study. In the future, multicentric studies are absolutely necessary in order to underpin further the role of ^18^F-FDG-PET as an additional method in clinical staging of patients with HL.
